# Outcomes of cytomegalovirus retinitis-related retinal detachment surgery in acquired immunodeficiency syndrome patients in an Asian population

**DOI:** 10.1186/1471-2415-14-150

**Published:** 2014-11-27

**Authors:** John X Wong, Elizabeth P Wong, Stephen C Teoh

**Affiliations:** Department of Ophthalmology, National Healthcare Group Eye Institute, Tan Tock Seng Hospital, Singapore, Singapore

**Keywords:** Retinal detachment, Cytomegalovirus retinitis, HIV, Surgery

## Abstract

**Background:**

This study reports the surgical outcomes of acquired immunodeficiency syndrome (AIDS) patients with Cytomegalovirus retinitis (CMVR) -related retinal detachments(RD) in an Asian population.

**Methods:**

Review of CMVR characteristics, surgical outcomes and complications in 19 eyes with CMVR-related RD that underwent surgery from January 2000 to June 2011.

**Results:**

CMVR was inactive in 73.7% of the eyes at time of surgery. Anatomical success was achieved in 14 eyes. Seven eyes (36.8%) had improvement of two or more lines in visual acuity (VA) and 8 eyes (42.1%) maintained VA. Thirteen eyes presented with worse than 6/120 vision, with 30.8% of them achieving ambulatory vision or better. Five eyes had re-detachments. Median durations from CMVR and immune recovery uveitis (IRU) diagnoses to RD were 2.7 and 1.0 months respectively.

**Conclusions:**

Surgery for CMVR-related RD is associated with good anatomical outcomes with most eyes maintaining or having improved vision. CMVR lesion size of <50% retinal area is associated with better outcomes. Eyes with CMVR and IRU require close monitoring for RD.

## Background

Cytomegalovirus retinitis (CMVR) is the most common opportunistic eye infection to occur in patients with acquired immune deficiency syndrome (AIDS) worldwide. It affects up to 22.1% of AIDS patients [1] even in this era of highly active anti-retroviral therapy (HAART). It results in visual morbidity from retinitis, or retinal detachment [2] (RD) that occurs in up to 24% of patients with CMVR [1].

There are limited reports from Asia of surgical outcomes for CMVR-related RD in AIDS patients. We report our surgical results of 19 patients with RD secondary to CMVR. The anatomical and functional outcomes of these patients were evaluated against patient, ocular and surgical factors.

## Methods

Medical records of HIV patients with CMVR-related RD who underwent retinal re-attachment surgery at our centre over 12 years from 1 January 2000 to 1 June 2011 were reviewed. These patients were under the primary care of infectious disease physicians from the Communicable Disease Centre (CDC), Singapore. Ethics approval was obtained from the National Healthcare Group (NHG) Domain Specific Review Board (DSRB) (reference A/10/515), in compliance with the Helsinki Declaration. Retinal re-attachment surgeries were performed by 6 experienced vitreo-retinal surgeons from the department.

The demographic characteristics, features of CMVR and RD were analyzed. Operative techniques for the majority of cases involved pars plana vitrectomy (20 or 23G), re-attachment by fluid-air exchange using a drainage retinotomy, endolaser, and internal tamponade with silicone oil or intraocular gas. Circumferential buckling and/or segmental scleral buckling were performed when indicated as judged by the surgeon-in-charge. Concurrent phacoemulsification was performed in eyes with significant cataracts. Proliferative vitreo-retinopathy (PVR) was classified based on the 1991 modification of the initial classification devised by the Retina Society [3].

Visual and anatomical outcomes of surgery and complications were assessed at three and six months after surgery. Visual acuity (VA) was measured using Snellen charts. For the purpose of data analysis, change of VA from light perception (PL) to hand movement (HM), from HM to counting fingers at two feet (CF), was defined as 2 line change in LogMAR vision. Anatomical success was defined as complete retinal re-attachment at six months without the need for repeat surgery. Functional success was defined as either preservation of VA post-operatively at better than ambulatory vision defined as BCVA of 6/120 or better, or improvement of 2 lines in LogMAR vision, after conversion from Snellen VA. All other post-operative complications were recorded. Elevated intraocular pressure (IOP) was defined as IOP > 25 mmHg.

CMVR was diagnosed clinically based on indirect ophthalmoscopy findings of the typical appearance of the disease. No aqueous or vitreous samples were obtained for viral PCR to confirm or exclude the diagnosis. The lesion typically consisted of an area of retinal necrosis or edema surrounded by granular infiltrates and a silvery-white border marking the edge of the active borders, with variable amounts of retinal hemorrhage and inflammatory vascular sheathing. Angiography was not performed. All cases of CMVR were diagnosed pre-operatively. CMVR lesions were described based on lesion size and their location. Lesion size was classified categorically as <25%, 25% to 49%, and >50% based on accurate clinical drawings. The location of CMVR lesion in each eye was categorized into three zones as described by Holland et al: Zone 1 was defined as the area within 1500 μm of the optic nerve or within 3000 μm of the centre of the macula. Zone 2 extended from zone 1 to the vortex veins and zone 3 involved the area anterior to the vortex veins [4]. For patients with CMVR involving multiple zones, the zone nearest to the macula was reported. CMV-related immune recovery uveitis (IRU) was defined and diagnosed clinically as a syndrome of anterior segment and vitreous inflammatory reactions directed towards CMV antigens in ocular tissues in patients with AIDS, associated with recovery of immunocompetence as a result of HAART [5]. The chronic inflammation from IRU is a risk factor for RD.

Intervals between CMVR and RD were expressed in months. Demographic information, ophthalmic examination and retinal detachment characteristics such as the presence of PVR, surgical characteristics including concurrent lens removal and CMVR activity were grouped into categories and analyzed as categorical variables against anatomical and functional success using Fisher’s exact test. Continuous variables such as CD4 counts were analyzed with Mann-Whitney U test when medians were compared. Data analyses were performed with IBM SPSS Statistics (Version 19, IBM Corp, New York, USA).

## Results

**Figure 1:**
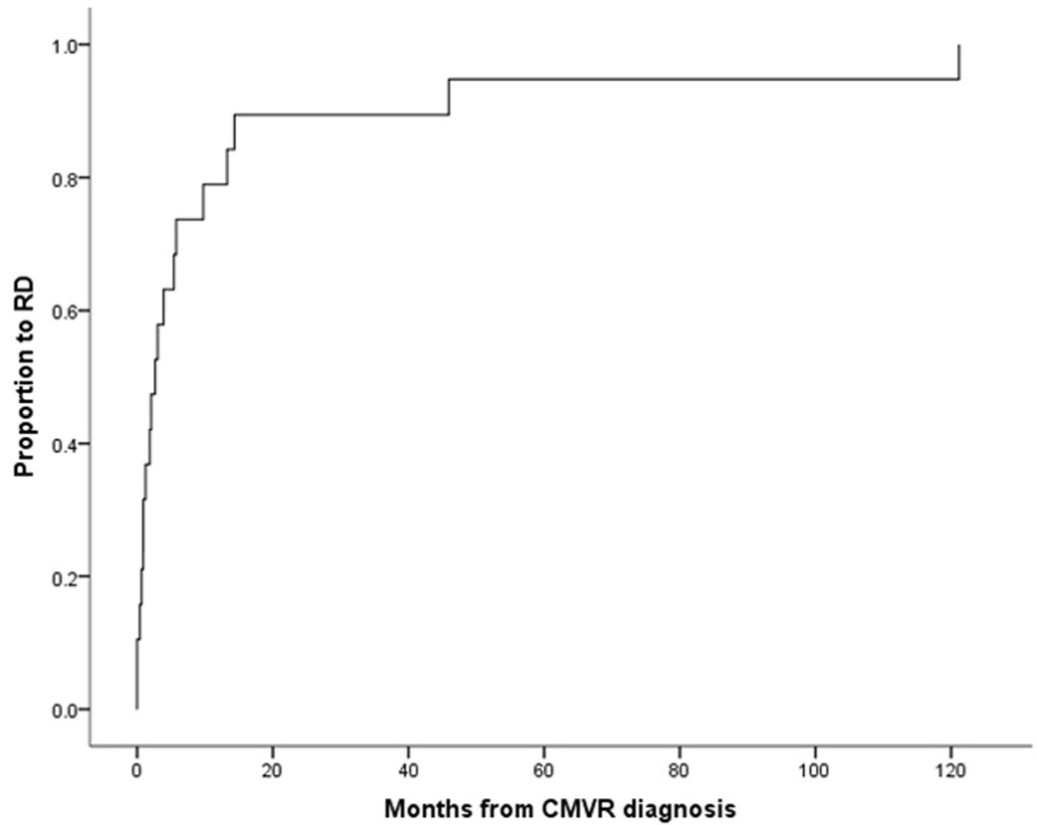
**Survival curve** - **time to RD from CMVR diagnosis.**

A total of 19 eyes with CMVR-related RD underwent retinal re-attachment surgery. These constituted 0.48% of 3996 patients who were diagnosed with HIV from years 2000-2011 in Singapore. Majority of our patients were males (84.2%) and of Chinese ethnicity (73.7%). Median CD4 count at time of diagnosis of RD was 91.5 cells/μL (range 15-571 cells/μL) with a median occurrence at 16.5 months (range 3.0-210.0 months) after HIV diagnosis. The median time from CMVR to RD was 2.7 months (range 0-121 months). Figure 1 shows the survival curve relating RD occurrence from CMVR onset. Eighteen (94.7%) patients were on HAART. Patient characteristics are summarized in Table 1.

**Table 1:** **Demographics and patient characteristics**

**Race,** **n (%)**	
Chinese	14 (73.7)
Malay	3 (15.8)
Others	2 (10.5)
**Gender,** **n (%)**	
Male	16 (84.2)
Female	3 (15.8)
**HAART use during RD,** **n (%)**	
Yes	18 (94.7)
No	1 (5.3)
**Intravitreal Ganciclovir use,** **n (%)**	
Yes	18 (94.7)
No^†^	1 (5.3)
**IRU before retinal detachment,** **n (%)**	
Yes	7 (36.8)
No	12 (63.2)
**Mean age,** **years** **(SD)**	
HIV diagnosis	40.6 (7.34)
Retinal reattachment surgery	45.6 (9.35)
**Median CD4 counts,** **cells** **/μL** **[Range]**	
HIV diagnosis	97.0 [2 - 830]
CMV retinitis diagnosis	53.0 [6 - 410]
Retinal detachment (RD)	91.5 [15 - 571]
Active CMVR at RD (n = 5)	67.0 [20 - 247]
Inactive CMVR at RD (n = 13)	93.0 [15 - 571]
**Median intervals,** **months** **[Range]**	
HIV to RD	16.5 [3 - 210]
CMV retinitis to RD	2.7 [0 - 121]
HIV to commencement of HAART	14.6 [0 - 217]
Commencement of HAART to RD	9.0 [-1 - 112]
Immune recovery uveitis to RD	1.0 [0 - 12]

^†^Received oral valganciclovir.

*HAART*: highly active anti-retroviral therapy, *RD*: retinal detachment, *IRU*: immune recovery uveitis, *HIV*: Human immunodeficiency virus, *CMVR*: cytomegalovirus retinitis.

Eighteen patients (94.7%) were treated with intravitreal ganciclovir upon diagnosis of CMVR with one patient receiving oral valganciclovir. This treatment was instituted immediately after the diagnosis of CMVR. Intravitreal ganciclovir was first given at an induction dose of 2 mg/0.04mls twice a week for 1 month and then maintained at 1 mg/0.02mls weekly until CD4 counts were above 100 cells/μL. CD4 counts were monitored 3 monthly when it was less than or equal to 500 cells/μL, and 6 monthly if greater than to 500 cells/ μL. Five eyes had active CMVR at the time of RD while the other eyes had quiescent disease after ganciclovir/valganciclovir treatment. The median CD4 count of patients with active disease at the time of RD was 67 cells/μL (range 20-247 cells/μL) compared to 93.0 cells/μL (range 15-57 1cells/μL) (p = 0.321) for patients with inactive CMVR. The characteristics of CMVR in these patients are summarized in Table 2. Seven (36.8%) patients had concomitant IRU, all of which occurred prior to RD. The median time from diagnosis of IRU to RD was 1.0 months (range 0-12 months). The median CD4 count of the 6 patients who had IRU just prior to retinal detachment was 154 cells/μL (range 20-333 cells/μL) compared to 68 cells/μL (range 15-571 cells/μL) in the patients who did not have IRU (p = 0.840).

**Table 2:** **Characteristics of CMV retinitis and retinal detachment**

	n	(%)
**CMV retinitis zonal involvement**		
Zone 1	3	(16.7)
Zone 2	10	(55.6)
Zone 3	5	(27.8)
**CMV retinitis size**		
< 25%	8	(44.4)
25 - 49%	6	(33.3)
50 - 74%	3	(16.7)
> = 75%	1	(5.6)
**CMV activity at RD**		
Active	5	(26.3)
Inactive	14	(73.7)
**Macula status of RD**		
Off	14	(73.7)
On	5	(26.3)
**Presence of PVR**		
Yes^†^	4	(21.1)
No	15	(78.9)
**Retinal defect**		
Hole	10	(52.6)
U-tear	6	(31.6)
No breaks found	3	(15.8)
**Area of retinal defect**		
CMV retinitis involved retina	15	(78.9)
Normal retina	1	(5.3)
None	3	(15.8)

^†^3 grade A PVR, 1 grade B/C.

*CMV*: cytomegalovirus, *RD*: retinal detachment, *PVR*: proliferative vitreo-retinopathy.

**Figure 2:**
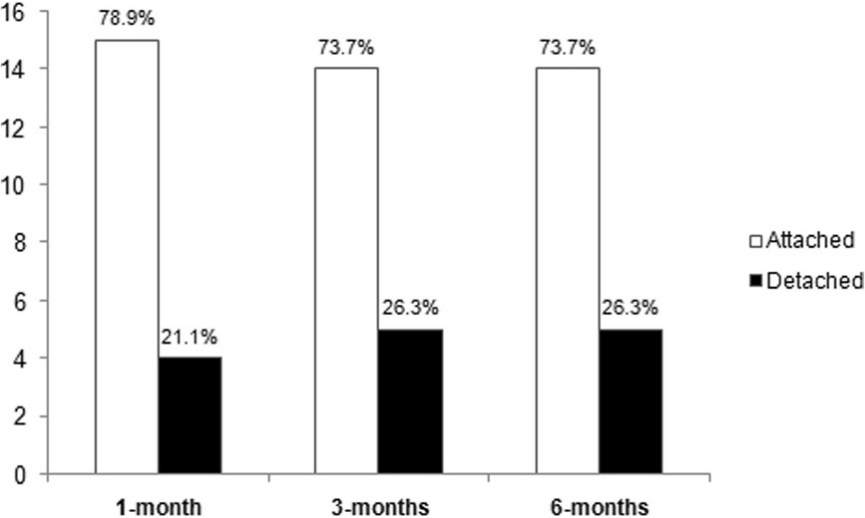
**Anatomical outcomes at one month, three months and six months post-surgery.**

**Figure 3:**
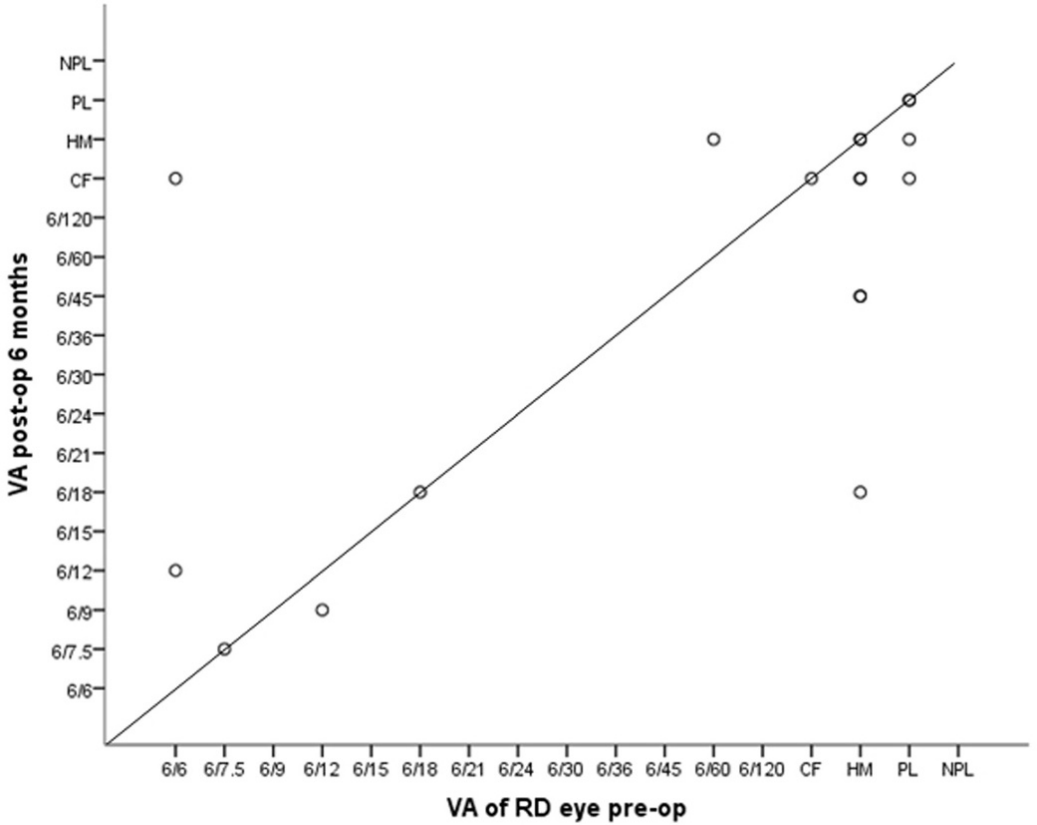
**Scatterplot of visual acuities at presentation (before surgery) and six months after surgery.**

The characteristics of the RDs and surgical details are summarized in Tables 2 and 3 respectively. Ten patients (52.6%) had a combined vitrectomy and scleral buckle procedure performed, seven patients (36.8%) underwent vitrectomy without scleral buckle, and 2 patients had only scleral buckling with cryopexy performed. Five patients had removal of silicone oil during the first 6 months after surgery. During follow-up, anatomical success was achieved in 14 of 19 patients (73.7%). Five patients (26.3%) had repeat detachments during the first six months of follow-up, two of whom declined further intervention due to poor prognosis. The anatomical outcomes at one month, three months and six months post-surgery are shown in Figure 2. Functional success was achieved in 10 patients (52.6%) at 3 and 6 months. At 6 months, 7 patients (36.8%) had improvement of VA by 2 or more lines, 8 patients (42.1%) retained similar VA, and three patients (15.8%) had drop of visual acuity by 2 or more lines. Of the 13 patients who presented with counting fingers or worse vision, 4 (30.8%) improved to 6/120 or better. Two patients had eventual BCVA of PL due to silicone oil-related glaucomatous optic neuropathy and retinal re-detachment respectively. Both of them declined further surgical intervention. Three patients had eventual NPL vision- one patient had repeat RD and declined surgery, the second suffered from optic atrophy and the third patient developed phthisis bulbi after retinal re-detachment. The VA outcomes are shown in Figure 3.

**Table 3:** **Summary of surgical characteristics and post**-**operative complications**

	n	(%)
**Concurrent cataract surgery**		
Yes^†^	6	(31.6)
No	13	(68.4)
**Buckling procedure**		
Yes	10	(52.6)
No	9	(47.4)
**Type of intraocular tamponade**		
None	2	(10.5)
SF6	1	(5.3)
C3F8	3	(15.8)
SO1300	8	(42.1)
SO5700	2	(10.5)
Heavy SO	3	(15.8)
**Post**-**op complications**		
Band keratopathy	2	(10.5)
High IOP > 25 mmHg	4	(21.1)
PVR	4	(21.1)
Optic atrophy (unspecified cause)	2	(10.5)
Cataract (11 phakic eyes after RD surgery)	6	(54.5)

^†^4 patients left aphakic, 2 had primary IOL implantation.

2 out of 4 aphakic patients underwent secondary *IOL* implantation, 1 patient had silicon oil glaucoma and was left aphakic, the other had poor vision and band keratopathy and declined further surgery.

*SO*: silicone oil, *IOP*: intraocular pressure, *PVR*: proliferative vitreo-retinopathy.

A further subset analysis was performed on patients who underwent vitrectomy. The outcomes of patients who underwent 20-gauge versus 23-gauge vitrectomy were compared. The anatomical success rates at 3 and 6 months of patients who underwent 23-gauge vitrectomy was 88.9% as compared to 62.5% in patients who underwent 20-gauge vitrectomy. The functional success rates were 77.8% and 12.5% for 23-gauge and 20-gauge vitrectomy respectively.

The complications of retinal re-attachment surgery in our patients are shown in Table 3. The most common complications were cataract formation, PVR and elevated IOP. Of the 11 phakic eyes post-RD surgery, six (54.5%) developed cataracts. The median time to development of cataract was six months (range 1 - 15 months).

The results of univariate analysis of factors affecting anatomical and functional success are shown in Table 4. Patients with <50% areas of CMVR showed a trend towards anatomical success (p = 0.197) and functional success (p = 0.245). There was also a trend towards anatomical success (p = 0.201) and functional success (p = 0.09) in patients who underwent 23-gauge vitrectomy. Active CMVR at the time of RD was not associated with higher re-detachment rates (p = 0.570) as compared to inactive CMV retinitis.

**Table 4:** **Univariate analysis of factors affecting anatomical success and functional success**

Characteristics	p-value*
**Anatomical success at 6 months vs**	
CD4 counts at RD	0.915
CMVR activity at surgery	0.570
CMV size </> 50%	0.197
Macula on/off RD	> 0.999
Presence of PVR	0.272
Presence of IRU	0.603
Concurrent scleral buckling	0.628
23G/20G vitrectomy	0.201
**Functional success at 6 months vs**	
CD4 counts at RD	0.847
CMV size </> 50%	0.103
CMVR activity at surgery	0.314
CMV macula involvement	0.088
RD size </> 50%	0.321
Macula on/off RD	> 0.999
Presence of PVR	> 0.999
Presence of IRU	0.367
23G/20G vitrectomy	0.090

*RD*: retinal detachment, *CMVR*: cytomegalovirus retinitis,

*PVR*: proliferative vitreo-retinopathy, IRU: immune recovery uveitis.

*CD4 counts at RD analysed with Mann Whitney test, other variables analysed with Fisher’s.

Exact test.

## Discussion

Retinal detachments still occur despite successful control of CMVR with anti-CMV medications. The risk of RD is increased with CMVR. Gliotic bands found at the border of normal and CMVR-affected retinae have been postulated to form as a response to inflammatory insult. This acts as a source of vitreo-retinal traction leading to retinal breaks and retinal RD [6]. Surgical re-attachment is necessary to preserve vision and improve the quality of life in these patients. Favorable treatment results of CMV-related RDs by vitrectomy and silicone oil injection over other techniques such as scleral buckling, pneumatic retinopexy or vitrectomy and fluid gas exchange have been reported for these complex detachments. Permanent silicone oil tamponade in the vitreous cavity have been advocated to seal current and future holes should retinitis progress, and also to provide a clear media for laser retinopexy. However techniques and preferences have varied over the years.

Anatomical success rates for CMV-related RD are reported to be lower because of the presence of multiple breaks, difficulty in identifying and sealing breaks within necrotic retinae, and the posterior location of breaks [7]. The anatomical success and ambulatory vision rates reported in current literature are 70-84% [6–10] and 65-84% [8, 11] respectively, in CMVR-related RD surgery. This is lower than results of primary rhegmatogenous RD repair which achieved anatomical success rates of 72-92% [12, 13]. Our anatomical and functional success rates were 73.7% and 52.6% respectively. Although prognosis after surgery for CMV-related RD is poor, up to half of these patients will regain ambulatory vision which contribute to overall better function and quality of life.

Factors related to better anatomical outcomes within our patients include a smaller lesion size (<50%), the use of 23-gauge vitrectomy systems and the absence of PVR. Smaller lesion size (<50%) and the use of 23-gauge vitrectomy systems was also related to better functional outcome in our patients. The trend towards better outcomes for patients who underwent 23-gauge vitrectomy as compared to patients who underwent 20-gauge vitrectomy strongly suggests that improvement in vitrectomy systems with time has a positive influence on patient outcomes. CD4 counts were found to be unrelated to the surgical outcomes in our patients. Median CD4 count for patients with active CMVR at the time of RD was lower than patients with inactive CMVR, consistent with higher risks of opportunistic infections in patients with lower CD4 count levels. Untreated CMVR spreads at an average of 250-350 μm per week [14] and even in cases where anti-CMV therapy has been commenced, prior to control of active retinitis, there is still progression of the disease albeit at a slower rate. It is thus likely that for patients with active retinitis at the time of surgery, the area of retinitis will continue to spread beyond areas of retinopexy resulting in new retinal breaks and recurrent detachments. As such it is prudent to include several rows of barrier retinopexy in cases of RD repair in the presence of active retinitis, more so than in standard cases of retinal breaks in rhegmatogenous RDs. Active CMVR was however not associated with a clinically higher risk of re-detachment among our patients.

IRU is a causative factor for PVR, the main risk factor in failed RDs, due to retinal traction [15]. Irvine *et al* reported that the mean times of CMVR to RD was 5.5 months for patients with more than three diopters of myopia, and 8 months in those with less than 1 diopter of myopia [16]. Our study revealed that a high proportion of patients had a short median interval of 2.7 months and 1.0 month between the occurrences of CMVR and IRU to the development of RD respectively. This suggests that patients need close monitoring for the development of RD even after anti-CMV therapy and HAART have been commenced.

Our results showed that apart from cataract formation (54.5%), complication rates were generally low. Cataract incidence after silicone oil tamponade has been reported to range between 30-100% [9, 16, 17] Our median onset time of cataract of six months was comparable to the published rates of four to seven months [8, 18, 19]. Clear lens extraction with intraocular lens implantation during repair of RD or during removal of silicone oil was advocated by Engstrom to improve long term visual rehabilitation and decrease morbidity associated with further surgeries [20]. Although concurrent cataract extraction during RD repair may allow better anterior vitreous clearance, we did not routinely perform lens extraction surgery unless indicated, due to greater post-operative intraocular inflammation with its attendant complications in these inflamed eyes, as well as the potential loss of accommodation in these patients who are frequently younger than those who present with primary rhegmatogneous RDs. Almost 30% of our patients were below 40 years old, and lens extraction surgery would have resulted in the loss of accommodation in these patients. Increased IOP is a common complication after vitrectomy and silicone oil tamponade. A transient ocular hypertension has been reported to occur in between 12-70% of cases, and development of glaucoma in up to 40% of cases with complex RDs [21, 22]. Our experience was similar with 21.1% of patients developing a transient IOP rise after surgery. This was higher than the 7.5% of patients who had transient IOP rise after surgery in our audit of patients who underwent surgical reattachment for primary rhegmatogenous RDs (unpublished data). Of the four patients with raised IOP after surgery, only one required treatment with topical IOP-lowering eyedrops for more than two months duration. None require glaucoma filtration surgery. Two patients (10.5%) developed band keratopathy after surgery, which was reported to occur in 4-6% of eyes with complex RD [8, 13]. Other reported complications include progressive optic atrophy and possible retinal toxicity due to both CMV infection and silicone oil toxicity [23].

We acknowledge that there are limitations in our study. Although this was a retrospective study, all our patients had at least 6 months of follow-up after initial surgery. We felt that this was an appropriate and adequate duration for visual rehabilitation, or time for development of any complications. Six vitreo-retinal surgeons were involved in the surgeries for these 19 eyes and this may lead to a study bias with regards to surgical outcomes. The operative techniques were however fairly standard and performed by trained and experienced surgeons. Subset analysis for 20 and 23-gauge vitrectomy was performed to account for changing surgical systems with time. Singapore is classified by WHO as a low-risk HIV epidemic country [24] with reasonably good accessibility to anti-CMV therapy and HAART. As such even though data was collated over 12 years, we had a relatively small number of patients. However this also reflects the low incidence of CMVR-related RDs in the HIV population locally. This may have affected our analysis as correlation between our outcomes and CMVR and RD characteristics did not yield statistically significant results although there was a statistical trend towards a difference. A study of a larger number of patients may increase the robustness of statistical analysis. Nonetheless, despite the smaller number of patients, the participants in this study were consecutive patients of all RD secondary to CMVR and had all relevant clinical information obtained, thus we felt represented a good sample of our population.

## Conclusion

AIDS patients with CMVR have a high risk of RD and surgery for these detachments have a good outcome to improve vision or preserve ambulatory vision. With advancements in anti-retroviral therapy, quality-of-life is of increasing importance to AIDS patients today who have a much longer life expectancy as compared to AIDS patients diagnosed with CMVR previously. Retinal re-attachment surgery for patients with CMVR-related RD gives them a viable chance of maintaining or improving their vision, and their quality of life.
